# The association between active cigarette smoking and acute gastrointestinal morbidity in US adults: A NHANES-based cross-sectional analysis

**DOI:** 10.1097/MD.0000000000048409

**Published:** 2026-04-24

**Authors:** Fang Yu, Ruili Wei, Haining Chen, Ming Qiu, Debin Huang

**Affiliations:** a The First Affiliated Hospital of Guangxi Medical University, Nanning, Guangxi, China.

**Keywords:** acute gastroenteritis, epidemiology, NHANES, public health, smoking

## Abstract

Acute gastroenteritis, characterized by vomiting and/or diarrhea, places a significant burden on healthcare systems and the economy. Although tobacco is recognized as a risk factor for chronic gastrointestinal (GI) diseases, its role in acute GI morbidity has not been widely investigated in population-based studies. To quantify the independent association between smoking status and 30-day prevalence of acute GI illness involving vomiting and/or diarrhea, adjusting for key confounders. We used nationally representative data from the 2005 to 2010 National Health and Nutrition Examination Survey (n = 12,358 adults aged ≥20 years) to classify smoking status as never, former, or current. The outcome was self-reported GI illness involving vomiting and/or diarrhea in the past 30 days. We employed staged multivariable logistic regression, sequentially adjusting for the following factors: demographics, comorbidities and body mass index, diet, and inflammatory and nutritional biomarkers. Current smokers had a significantly higher prevalence of GI illness (11.3%) than never smokers (7.5%) and former smokers (8.2%) (*P* < .001). In the fully adjusted models, current smoking was associated with a 52% increase in the odds of GI illness (odds ratio: 1.52; 95% confidence interval: 1.29–1.79; *P* < .001), whereas former smokers had odds that were 19% higher (odds ratio: 1.19; 95% confidence interval: 1.01–1.41; *P* = .037). Stratified analyses confirmed consistency across demographic and clinical subgroups (all *P*-interactions > .05). Active cigarette smoking is an independent risk factor for acute GI morbidity. The reduced risk among former smokers suggests that the harm caused by tobacco can be reversed, thus supporting smoking cessation as a preventive strategy. This study broadens our understanding of smoking-related diseases and provides insights into integrated tobacco control interventions.

## 1. Introduction

Tobacco use is the leading preventable cause of disease and premature death worldwide.^[1,2]^ Its harmful effects extend significantly beyond the respiratory and cardiovascular systems.^[3]^ The gastrointestinal (GI) tract is a critical yet often overlooked target, acting as a primary interface and active participant in the systemic response to cigarette smoke.^[4]^ Mechanistic studies indicate that tobacco constituents impair GI homeostasis through multiple pathways,^[5,6]^ including delayed gastric emptying,^[7]^ compromised mucosal blood flow,^[8]^ disrupted acid-base equilibrium,^[9]^ suppression of innate and adaptive immunity,^[10]^ and profound alterations to the structure and function of the gut microbiota.^[11]^ Taken together, these effects are hypothesized to lower the threshold for the development of various GI pathologies, ranging from acute conditions such as enteric infections to chronic diseases such as peptic ulcers and inflammatory bowel disease.^[12]^

Epidemiological evidence reveals a complex and sometimes paradoxical relationship between smoking and specific GI disorders.^[13]^ Active smoking is consistently associated with an increased incidence and severity of Crohn, yet it appears to offer some protection against ulcerative colitis.^[14]^ Smoking has also been linked to impaired ulcer healing, increased postsurgical complications, and recurrent peptic disease.^[15]^ Beyond these well-documented chronic effects, tobacco smoke may acutely compromise intrinsic GI defense mechanisms.^[8]^ This is achieved by disrupting epithelial tight junctions, altering mucosal immune signaling, and fostering a dysbiotic microbiota that is skewed towards pro-inflammatory or pathogenic species.^[16]^ Consequently, smoking could plausibly increase vulnerability to acute GI insults and exacerbate symptoms, such as vomiting and diarrhea.^[17]^ However, a significant knowledge gap remains; robust, population-based evidence is lacking. Previous investigations have focused primarily on pediatric cohorts, secondhand exposure, and specific pathogens, yielding inconclusive results.^[18–20]^ Large-scale, nationally representative studies that can comprehensively account for sociodemographic, clinical, dietary, and inflammatory confounders are lacking.

The National Health and Nutrition Examination Survey (NHANES) is an unparalleled resource for addressing this issue.^[21]^ Its integrated design combines rigorous data collection on smoking behavior and recent GI symptoms via standardized recall with extensive covariate information and objective laboratory biomarkers. This allowed for a robust assessment of potential associations while controlling for key confounders. This cross-sectional study used data from the 2005 to 2010 cycles and aimed to quantify the independent association between current cigarette smoking and the 30-day prevalence of acute GI illness characterized by vomiting or diarrhea among US adults. We hypothesized that current smoking is positively associated with this outcome. Furthermore, we sought to determine whether former smoking confers residual risk and assessed the consistency of the associations across diverse demographic and clinical subgroups.

## 2. Materials and methods

### 2.1. Data sources and study population

**Figure 1.**
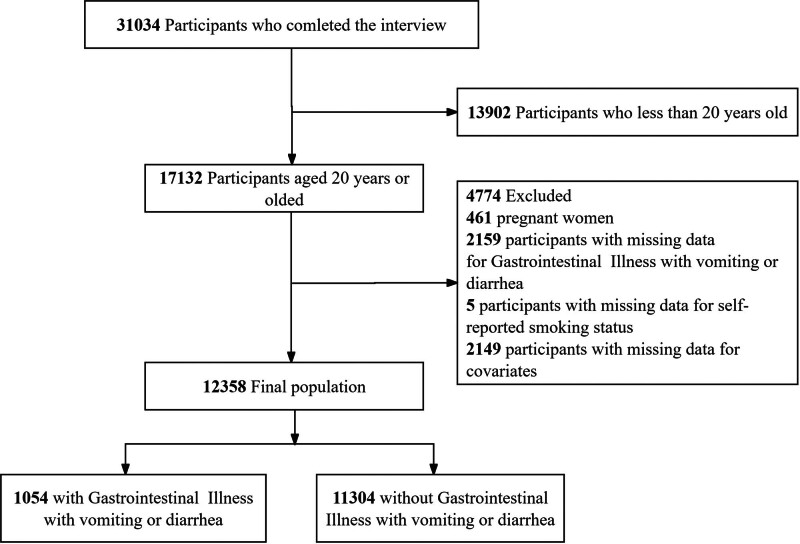
The flowchart of participant inclusion and exclusion.

This analysis used cross-sectional data from 3 consecutive cycles (2005–2006, 2007–2008, and 2009–2010) of the NHANES. The NHANES uses a complex, multistage, stratified probability sampling design to produce estimates that are representative of the entire US civilian, noninstitutionalized population. The National Center for Health Statistics (NCHS) has documented the survey methodology in detail, including protocols for data collection and sampling weights.^[22,23]^ All participants provided written informed consent and the survey procedures were approved by the NCHS Research Ethics Review Board. Our analysis was restricted to adults aged ≥20 years who completed the household interview and provided valid responses to the smoking history and GI symptom questionnaires during 2005–2010. Pregnant individuals were excluded because of the potential confounding factors of physiological changes and pregnancy-specific nausea and diarrhea. Participants with missing data on smoking status, GI outcomes, or essential covariates were also excluded, resulting in an analytical sample of 12,358 individuals. Figure 1 shows the flowchart of participant selection.

### 2.2. Assessment of smoking exposure

Smoking status was defined based on responses to a standardized self-report questionnaire. The participants were classified into 1 of 3 mutually exclusive categories^[24]^:

*Never smokers*: having smoked fewer than 100 cigarettes in their lifetime.
*Former smokers*: reported having smoked at least 100 cigarettes but were not currently smoking at the time of the survey.
*Current smokers*: reported having smoked at least 100 cigarettes and currently smoking ``some days’’ or ``every day.’’

### 2.3. Outcome definition: acute GI illness

The primary outcome was defined as a self-reported episode of stomach or intestinal illness accompanied by vomiting or diarrhea within the previous 30 days. This was assessed using the specific NHANES question: “During the past 30 days, have you had a stomach or intestinal illness with vomiting or diarrhea?” Responses were dichotomized (yes/no) to focus on capturing recent acute symptomatic events rather than chronic conditions.

### 2.4. Covariate selection and measurement

Potential confounding variables were selected based on causal diagrams (directed acyclic graphs) and informed by prior literature and subject-matter knowledge.^[25–28]^ These factors are potentially associated with both smoking behavior and risk of GI symptoms. Covariates included the following:

*Demographics*: Age (continuous variable in years), sex (male or female), race/ethnicity (non-Hispanic white, non-Hispanic black, Mexican American, or other), educational attainment (categorical variable: less than 9 years; 9 to 12 years; or more than 12 years), marital status (married or living with a partner; living alone), and family income categorized by poverty-income ratio (low, medium, or high, according to NHANES definitions).^[26]^
*Comorbidities*: Self-reported, physician-diagnosed history of hypertension, diabetes mellitus, coronary heart disease, stroke, or any cancer/malignancy; each binary variable: yes/no).
*Anthropometrics and laboratory biomarkers*: Body mass index (BMI; kg/m^2^, continuous), serum C-reactive protein (CRP; mg/L, continuous marker of systemic inflammation), serum potassium (continuous; mmol/L), and serum sodium (continuous; mmol/L).
*Dietary factors*: Use of dietary supplements (yes/no); mean daily intake estimates derived from 24-hour dietary recalls for total energy (kcal), total fat (g), total protein (g), and total carbohydrate (g).

### 2.5. Statistical analysis

All statistical analyses were performed using R software (version 4.2), with data management aided by Free Statistics software (version 2.3, Beijing, China). To account for the complex survey design involving strata, primary sampling units, and interview weights, sample weights were incorporated into all analyses to yield nationally representative estimates with correct standard errors.

#### 2.5.1. Descriptive statistics

Participant characteristics were summarized overall and according to smoking status. Continuous variables were presented as the mean ± standard deviation if they were normally distributed or as the median with the interquartile range if they were skewed (normality was assessed using the Shapiro–Wilk test, *P* < .05). Categorical variables were presented as frequencies and weighted proportions. Group comparisons across smoking categories were performed using the appropriate tests: Student *t* test or Mann–Whitney *U*-test was used for continuous variables, and a Chi-squared test was used for categorical variables (Table 1).

**Table 1:** Baseline characteristics of participants (NHANES 2005–2010).

Variables	Total (n = 12,358)	Self-reported smoking status, n (%)			*P*
		Never (n = 6413)	Former (n = 3195)	Current (n = 2750)	
Sex, n (%)					<.001
Male	6257 (50.6)	2733 (42.6)	1965 (61.5)	1559 (56.7)	
Female	6101 (49.4)	3680 (57.4)	1230 (38.5)	1191 (43.3)	
Age (yr)	49.9 ± 17.8	48.2 ± 18.0	58.3 ± 16.5	44.0 ± 15.1	<.001
Race/ethnicity, n (%)					<.001
Non-Hispanic white	6337 (51.3)	2901 (45.2)	1956 (61.2)	1480 (53.8)	
Non-Hispanic black	2376 (19.2)	1303 (20.3)	470 (14.7)	603 (21.9)	
Mexican American	2179 (17.6)	1327 (20.7)	480 (15)	372 (13.5)	
Others	1466 (11.9)	882 (13.8)	289 (9)	295 (10.7)	
Education level (yr), n (%)					<.001
<9	1408 (11.4)	726 (11.3)	400 (12.5)	282 (10.3)	
9–12	4929 (39.9)	2225 (34.7)	1190 (37.2)	1514 (55.1)	
>12	6021 (48.7)	3462 (54)	1605 (50.2)	954 (34.7)	
Marital status, n (%)					<.001
Married or living with partners	7592 (61.4)	3950 (61.6)	2158 (67.5)	1484 (54)	
Living alone	4766 (38.6)	2463 (38.4)	1037 (32.5)	1266 (46)	
Family income, n (%)					<.001
Low	3626 (29.3)	1692 (26.4)	737 (23.1)	1197 (43.5)	
Medium	4754 (38.5)	2473 (38.6)	1315 (41.2)	966 (35.1)	
High	3978 (32.2)	2248 (35.1)	1143 (35.8)	587 (21.3)	
Hypertension, n (%)					<.001
No	8772 (71.0)	4694 (73.2)	1950 (61)	2128 (77.4)	
Yes	3586 (29.0)	1719 (26.8)	1245 (39)	622 (22.6)	
Diabetes, n (%)					<.001
No	10,962 (88.7)	5726 (89.3)	2701 (84.5)	2535 (92.2)	
Yes	1396 (11.3)	687 (10.7)	494 (15.5)	215 (7.8)	
Coronary heart disease, n (%)					<.001
No	11,837 (95.8)	6226 (97.1)	2946 (92.2)	2665 (96.9)	
Yes	521 (4.2)	187 (2.9)	249 (7.8)	85 (3.1)	
Cancer/malignancy, n (%)					<.001
No	11,166 (90.4)	5887 (91.8)	2718 (85.1)	2561 (93.1)	
Yes	1192 (9.6)	526 (8.2)	477 (14.9)	189 (6.9)	
Stroke, n (%)					<.001
No	11,902 (96.3)	6244 (97.4)	3009 (94.2)	2649 (96.3)	
Yes	456 (3.7)	169 (2.6)	186 (5.8)	101 (3.7)	
Dietary supplements taken, n (%)					<.001
No	6357 (51.4)	3181 (49.6)	1339 (41.9)	1837 (66.8)	
Yes	6001 (48.6)	3232 (50.4)	1856 (58.1)	913 (33.2)	
Calorie consumption (kcal/d), mean ± SD	2113.4 ± 1009.1	2035.9 ± 936.8	2083.7 ± 916.9	2328.8 ± 1221.5	<.001
Fat consumption (g/d), mean ± SD	79.3 ± 46.7	76.1 ± 43.8	80.2 ± 44.6	85.6 ± 54.3	<.001
Protein consumption (g/d), mean ± SD	81.3 ± 43.1	79.4 ± 40.9	81.4 ± 39.9	85.5 ± 50.8	<.001
Carbohydrate consumption (g/d), mean ± SD	256.1 ± 126.4	252.1 ± 118.0	246.5 ± 113.2	276.9 ± 154.6	<.001
Body mass index (kg/m^2^), mean ± SD	29.0 ± 6.7	29.3 ± 6.9	29.5 ± 6.3	27.8 ± 6.6	<.001
C-reactive protein (mg/dL), median (IQR)	0.2 (0.1, 0.5)	0.2 (0.1, 0.4)	0.2 (0.1, 0.5)	0.2 (0.1, 0.5)	<.001
Potassium (mmol/L), mean ± SD	4.0 ± 0.3	3.9 ± 0.3	4.0 ± 0.4	4.0 ± 0.3	<.001
Sodium (mmol/L), mean ± SD	139.2 ± 2.3	139.2 ± 2.2	139.3 ± 2.5	139.1 ± 2.3	<.001
GI illness with vomiting or diarrhea, n (%)					<.001
No	11,304 (91.5)	5932 (92.5)	2932 (91.8)	2440 (88.7)	
Yes	1054 (8.5)	481 (7.5)	263 (8.2)	310 (11.3)	

GI = gastrointestinal, IQR = interquartile range, NHANES = National Health and Nutrition Examination Survey, SD = standard deviation.

#### 2.5.2. Crude associations

Univariate logistic regression models were used to assess the unadjusted relationship between each potential predictor (covariate or smoking status) and outcome (GI illness involving vomiting or diarrhea) without adjustment. The results are reported in Table 2 as odds ratios (ORs) and 95% confidence intervals (CIs).

**Table 2:** The crude associations with GI illness involving vomiting or diarrhea.

Variable	OR (95% CI)	*P*-value
Sex		
Male	1 (reference)	
Female	1.49 (1.31–1.69)	<.001
Age (yr)	1 (0.99–1)	.102
Race/ethnicity		
Non-Hispanic white	1 (reference)	
Non-Hispanic black	0.98 (0.83–1.17)	.845
Mexican American	1.14 (0.96–1.34)	.141
Others	0.99 (0.81–1.22)	.943
Education level (yr)		
<9	1 (reference)	
9–12	0.99 (0.81–1.23)	.951
>12	0.95 (0.77–1.17)	.643
Marital status		
Married or living with partners	1 (reference)	
Living alone	1.29 (1.13–1.46)	<.001
Family income		
Low	1 (reference)	
Medium	0.74 (0.64–0.86)	<.001
High	0.59 (0.51–0.70)	<.001
Hypertension	1.43 (1.25–1.63)	<.001
Diabetes	1.36 (1.14–1.63)	.001
Coronary heart disease	1.23 (0.92–1.64)	.14
Cancer	1.32 (1.08–1.60)	.006
Stroke	1.66 (1.26–2.20)	<.001
Body mass index (kg/m^2^)	1.02 (1.01–1.03)	<.001
Dietary factors		
Dietary supplement use	0.88 (0.77–1)	.047
Total energy intake	1 (1–1)	.063
Fat intake	1 (1–1)	.039
Protein intake	1 (1–1)	<.001
Carbohydrate intake	1 (1–1)	.308
C-reactive protein (mg/dL)	1.27 (1.2–1.35)	<.001
Potassium (mmol/L)	0.76 (0.63–0.92)	.005
Sodium (mmol/L)	0.97 (0.94–0.99)	.016
Self-reported smoking status		
Never	1 (reference)	
Former	1.11 (0.95–1.29)	.207
Current	1.57 (1.35–1.82)	<.001

GI = gastrointestinal, OR = odds ratio.

#### 2.5.3. Multivariable regression modeling

Four sequential logistic regression models were constructed to isolate the independent association of smoking status and assess the influence of confounding factors:

*Model 1*: unadjusted.
*Model 2*: adjusted for sex, age, race/ethnicity, education level, marital status, and family income.
*Model 3*: adjusted for Model 2 variables plus hypertension, diabetes, coronary heart disease, stroke, cancer, and BMI.
*Model 4*: adjusted for Model 3 variables plus dietary supplement use, total energy, fat, protein, carbohydrate, CRP, potassium, and sodium.

#### 2.5.4. Sensitivity and subgroup analyses

**Figure 2.**
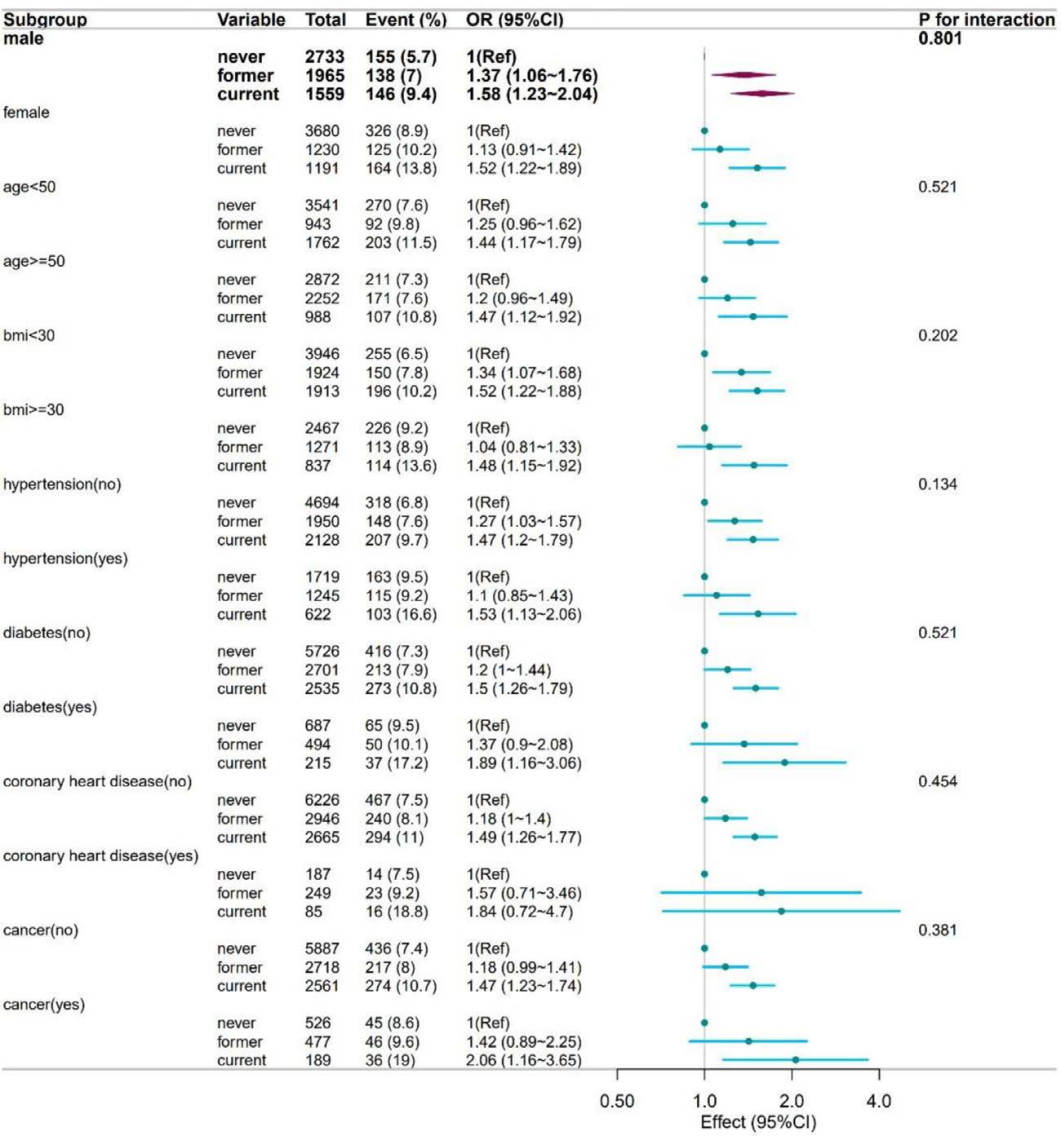
The subgroup analysis of the association between current smoking and GI illness with vomiting or diarrhea. Forest plot illustrating adjusted odds ratios (ORs) and 95% confidence intervals (CIs) for associations within each subgroup. All point estimates exceed 1, and the confidence intervals exclude the null value, indicating a statistically significant positive association within each stratum. The *P*-values for the interaction tests were not significant. GI = gastrointestinal.

To evaluate the robustness of the primary association (current vs never smokers) and explore potential effect modification, stratified analyses were performed using the fully adjusted model (Model 4) within the following subgroups: sex, age (under 50 vs 50 and over), BMI (under 30 vs 30 and over), and presence or absence of hypertension, diabetes, coronary heart disease, and cancer. The interaction on the multiplicative scale was formally tested using Wald tests for the product term (smoking status × subgroup variable). The results are summarized in a forest plot (Fig. 2).

### 2.6. Ethics statement

The NHANES survey protocol was approved by the NCHS Research Ethics Review Board. Written informed consent was obtained from all participants prior to data collection. As this study used anonymized, publicly available NHANES data, further approval from the institutional review board was not necessary.

## 3. Results

### 3.1. Participant characteristics

Table 1 details the baseline characteristics of the 12,358 participants, both overall and stratified by smoking status. Significant differences were observed across all domains (*P* < .001). There was a clear age gradient: current smokers were the youngest (mean age 44.0 years), followed by never smokers (mean age 48.2 years), and former smokers were the oldest (mean age 58.3 years). Males constituted a higher proportion of current smokers (56.7%) than of never (45.8%) or former (47.9%) smokers. Current smokers had the lowest educational attainment (only 34.7% had >12 years of education), in contrast to never (52.1%) and former (48.3%) smokers. Socioeconomic disadvantage was more prevalent among current smokers: 46.0% lived alone and 43.5% were in the lowest income bracket. The prevalence of comorbidities reflected the age distribution: hypertension was least prevalent in current smokers (22.6%), more prevalent in never smokers (28.9%), and most prevalent in former smokers (42.7%). A similar pattern was seen for diabetes (7.8%, 8.9% and 17.4% respectively). The mean BMI was slightly lower in current smokers (27.8 kg/m^2^) than in never (29.0 kg/m^2^) or former (29.3 kg/m^2^) smokers. Current smokers reported different dietary patterns, with higher mean daily intakes of energy (2328 kcal), fat (85.6 g), and protein (85.5 g), but lower use of dietary supplements (33.2%) than never or former smokers (47–49%). Most importantly, the crude 30-day prevalence of the outcome (GI illness with vomiting/diarrhea) was significantly higher in current smokers (11.3%) than in never smokers (7.5%) or former smokers (8.2%; *P* < .001).

### 3.2. Unadjusted associations

The results of univariate logistic regression analysis are shown in Table 2. The following factors were significantly associated with an increased likelihood of reporting GI illness (*P* < .05): being female, living alone, having a low family income, hypertension, diabetes, stroke, cancer, higher BMI, greater daily protein intake, elevated serum CRP, and lower serum potassium and sodium levels. Current smokers exhibited substantially higher odds than never smokers (OR: 1.57; 95% CI: 1.35–1.82; *P* < .001), while former smokers showed a nonsignificant trend towards an increased risk (OR: 1.11; 95% CI: 0.95–1.29; *P* = .207). Age; race/ethnicity; educational attainment; coronary heart disease; use of dietary supplements; and total energy, fat, or carbohydrate intake were not significantly associated with the outcome.

### 3.3. Independent association of smoking status

The results of multivariable logistic regression analysis are shown in Table 3. In the unadjusted model, current smokers had significantly higher odds of GI illness than never smokers (OR: 1.57; 95% CI: 1.35–1.82; *P* < .001). Adjustment for core demographic factors (age, sex, and race/ethnicity; Model 2) had only a minimal effect on this association (OR: 1.59; 95% CI: 1.36–1.86; *P* < .001). Including socioeconomic status and clinical comorbidities (including BMI; Model 3) produced a similar OR (1.62; 95% CI: 1.38–1.90; *P* < .001). Importantly, the association remained strong in the fully adjusted model (Model 4), which incorporated dietary variables and biomarkers. Current smokers were found to have 52% higher odds of GI illness (OR: 1.52; 95% CI: 1.29–1.79; *P* < .001). Former smokers initially showed a borderline association in the unadjusted model, which became statistically significant after adjusting for demographic and socioeconomic factors (Model 2: OR: 1.24; 95% CI: 1.05–1.46; *P* = .010) and remained significant in the full model, indicating a 19% increased risk (OR: 1.19; 95% CI: 1.01–1.41; *P* = .037). This gradient of risk (current smokers > former smokers > never smokers) supports a dose–response relationship, which is consistent with a potential causal effect of smoking.

**Table 3:** The multivariable associations between smoking status and GI illness with vomiting or diarrhea.

Variable	GI illness with vomiting or diarrhea							
	Model 1		Model 2		Model 3		Model 4	
	OR (95% CI)	*P*-value	OR (95% CI)	*P*-value	OR (95% CI)	*P*-value	OR (95% CI)	*P*-value
Self-reported smoking status								
Never	1 (Ref)		1 (Ref)		1 (Ref)		1 (Ref)	
Former	1.11 (0.95–1.29)	.207	1.24 (1.05–1.46)	.01	1.2 (1.01–1.41)	.034	1.19 (1.01–1.41)	.037
Current	1.57 (1.35–1.82)	<.001	1.59 (1.36–1.86)	<.001	1.62 (1.38–1.90)	<.001	1.52 (1.29–1.79)	<.001

*Model 1*: unadjusted. *Model 2*: adjusted for sex, age, race/ethnicity, education level, marital status, and family income. *Model 3*: adjusted for Model 2 variables plus hypertension, diabetes, coronary heart disease, stroke, cancer, and BMI. *Model 4*: adjusted for Model 3 variables plus dietary supplement use, total energy, fat, protein, carbohydrate, C-reactive protein, potassium, and sodium.

BMI = body mass index, GI = gastrointestinal, OR = odds ratio.

### 3.4. Robustness and consistency

The subgroup analyses demonstrated the robustness and generalizability of the primary findings (Fig. 2). A positive association between current smoking and GI illness was consistently observed across all predefined subgroups, including sex, age group (<50 or ≥50 years), BMI category (<30 or ≥30 kg/m^2^), and the presence or absence of hypertension, diabetes, coronary heart disease, and cancer. Formal tests for interactions were not significant for all subgroup variables (all *P*-interactions > .05). This finding suggests that the increased risk of acute GI morbidity associated with current smoking remains consistent across different population groups, thereby strengthening the external validity of our findings.

## 4. Discussion

This large, nationally representative study provides robust evidence that smoking cigarettes is independently associated with a significantly higher prevalence of acute GI illness characterized by vomiting or diarrhea among US adults. This association remained consistent even after adjusting for a wide range of potential confounders, including demographics, socioeconomic status, comorbidities, anthropometrics, dietary patterns, and systemic inflammatory/nutritional biomarkers. The increased risk was substantial (52% higher odds for current smokers) and consistent across the various demographic and clinical subgroups. Furthermore, we observed a gradient of risk, with former smokers exhibiting a modest but significant residual elevation in risk (19% higher odds) compared to never-smokers. Although tobacco is a well-established risk factor for numerous chronic GI diseases,^[12,29]^ this study addresses a critical gap in the literature by demonstrating its link to acute nonspecific GI morbidity in the general adult population. Our findings significantly expand the spectrum of health consequences attributable to cigarette smoking.

Several interlinked biological mechanisms offer plausible explanations for this association.^[30,31]^ First, smoking induces broad immunosuppression, impairing both innate and adaptive immune functions.^[32]^ This reduces mucosal antibody production (e.g., secretory immunoglobulin A) and alters cytokine profiles, resulting in a less effective antipathogen response.^[33]^ This compromised immunity increases susceptibility to enteric pathogens and delays their clearance.^[17]^ Second, tobacco smoke profoundly alters the gut microbiome, reducing its overall diversity and fostering a dysbiotic state enriched in proinflammatory taxa or potential pathogens.^[11,16,34]^ Importantly, smoking cessation rapidly reversed many of these microbial changes, suggesting a direct effect of tobacco constituents.^[35]^ Such dysbiosis can erode colonization resistance, enabling pathogen overgrowth and promoting mucosal inflammation that amplifies diarrheal symptoms.^[17]^ Third, oxidative stress and inflammatory mediators present in tobacco smoke can directly damage the intestinal epithelial barrier by disrupting tight junctions and increasing permeability.^[16,36,37]^ This ``leaky gut’’ can allow luminal antigens, toxins, or microbes to translocate, potentially triggering inflammation and symptoms.^[38]^ Finally, nicotine and other tobacco components modulate GI motility and secretion by affecting the enteric nervous system and hormonal pathways.^[39]^ This could manifest as delayed gastric emptying (contributing to nausea and vomiting) or accelerated transit/secretory diarrhea.^[40]^ Taken together, these pathways (immune dysfunction, dysbiosis, barrier disruption, and neuromodulation) provide a coherent mechanistic framework that supports the observed epidemiological link.^[41]^

The clinical implications of these findings are significant. Although acute GI illness is usually self-limiting,^[42]^ it can lead to significant dehydration and increased utilization of healthcare services, as well as cause work absenteeism and place an economic burden on society.^[43–45]^ In vulnerable populations, such as infants, the elderly, and immunocompromised individuals, they can be life-threatening.^[46–48]^ Our findings highlight acute GI morbidity as an underrecognized extrapulmonary consequence of tobacco use.^[49]^ Clinicians should actively inquire about smoking status when evaluating patients presenting with acute vomiting or diarrhea, and should incorporate smoking cessation counseling as a key preventive strategy.^[50,51]^ From a public health perspective, tobacco control efforts aimed at reducing smoking prevalence could mitigate the population burden of acute GI illness and yield additional benefits.^[52–54]^

Our results are supported by several key findings. These include a large, nationally representative sample size, the use of standardized protocols for exposure and covariate assessment, a comprehensive sequential adjustment strategy to mitigate confounding factors and explore potential mediation pathways, and the application of complex survey methods to ensure accurate population-level estimates. Internal validity was further enhanced by consistent findings across sensitivity analyses and diverse subgroups.

The limitations of the study design and data must be acknowledged. The cross-sectional nature of this study precludes definitive conclusions about causality. While the observed risk gradient (current smokers > former smokers > never smokers) suggests that this is not the primary explanation, reverse causation (recent GI illness prompting changes in smoking behavior) cannot be entirely ruled out. The outcome relied on self-reporting of symptoms within a 30-day recall period, which lacks etiological specificity and introduces the potential for recall bias and misclassification of outcomes. Despite exhaustive covariate adjustment, residual confounding by unmeasured or imperfectly measured factors remains possible (e.g., detailed alcohol consumption, illicit drug use, recent travel history, occupational exposures, specific food-handling practices, psychological stress, and over-the-counter medication use). Dietary intake was assessed using a single 24-hour recall, which may not reliably capture dietary patterns relevant to GI health or infection risk. Finally, while systemic inflammation was captured via CRP, NHANES lacks data on gut-specific immunity, mucosal biomarkers, and stool diagnostics, which could provide deeper mechanistic insights.^[55]^

Future research should prioritize prospective cohort studies that measure smoking behavior repeatedly (using biomarkers such as cotinine to confirm this),^[25,56]^ laboratory-confirmed enteric infections, longitudinal microbiome profiling, and assessments of intestinal barrier function (using serum biomarkers such as zonulin or intestinal fatty acid-binding protein to confirm this),^[57–59]^ and mucosal immunity.^[60]^ Randomized controlled trials of smoking cessation interventions with GI illness as a prespecified endpoint are needed to establish whether quitting smoking reduces the incidence or severity of acute GI morbidity. Furthermore, mechanistic studies utilizing animal models or in vitro systems are essential to determine the precise molecular pathways by which tobacco smoke constituents compromise the gut barrier integrity and host defense.

## 5. Conclusion

This large, nationally representative study demonstrates a strong and independent association between current cigarette smoking and a significantly higher prevalence of acute GI illness presenting with vomiting or diarrhea among adults in the US. Former smokers also had a moderately elevated risk. These findings establish acute GI morbidity as a novel, clinically relevant consequence of tobacco use in addition to its well-known chronic effects. The observed risk gradient (current smokers > former smokers > never smokers) and the residual risk in former smokers emphasize the potential GI benefits of quitting smoking. These results reinforce the value of tobacco control programs and cessation support not only in preventing cancer, cardiovascular disease, and chronic respiratory disease, but also in potentially reducing the burden of acute GI illness. Further longitudinal and experimental studies are required to confirm the causality and elucidate the underlying biological mechanisms.

## Acknowledgments

We would like to express our gratitude to Jie Liu, from the Department of Vascular and Endovascular Surgery at the Chinese PLA General Hospital, for his valuable contributions to the statistical analysis and design of the study and for his critical review of the manuscript. We would also like to thank the Free Statistics team in Beijing, China for their technical support and data analysis tools.

## Author contributions

**Conceptualization:** Debin Huang.

**Data curation:** Fang Yu, Haining Chen, Ming Qiu.

**Formal analysis:** Ruili Wei, Debin Huang.

**Investigation:** Fang Yu, Haining Chen.

**Methodology:** Fang Yu, Ruili Wei, Haining Chen, Ming Qiu.

**Project administration:** Debin Huang.

**Resources:** Ming Qiu.

**Software:** Fang Yu, Debin Huang.

**Supervision:** Debin Huang.

**Visualization:** Ruili Wei.

**Writing – original draft:** Fang Yu, Haining Chen, Ming Qiu.

**Writing – review & editing:** Fang Yu, Ruili Wei, Ming Qiu, Debin Huang.
